# Academy of Medicine Notes

**Published:** 1893-12

**Authors:** 


### Academy of Medicine Notes.

THE next regular meeting of the Academy will be held Tuesday evening, December 5, 1893, when the following program will be carried out: Prostatic Diseases, with special reference to the Prostate of Old Age, Dr. Marcell Hartwig. Discussion by Dr. William S. Tremaine and Dr. William H. Heath.

